# Supplementary material for: BLTR1 Is Decreased in Steroid Resistant Pro-Inflammatory CD28nullCD8+ T Lymphocytes in Patients with COPD—The Spillover Hypothesis Explained?
Source: Biology (Basel). 2023 Sep 20;12(9):1261. doi: 10.3390/biology12091261 (PMC10525726; doi:10.3390/biology12091261)
Supplement: Supplementary file 1 [file biology-12-01261-s001.zip › biology-2578427-supplementary.pptx]

## Slide 1
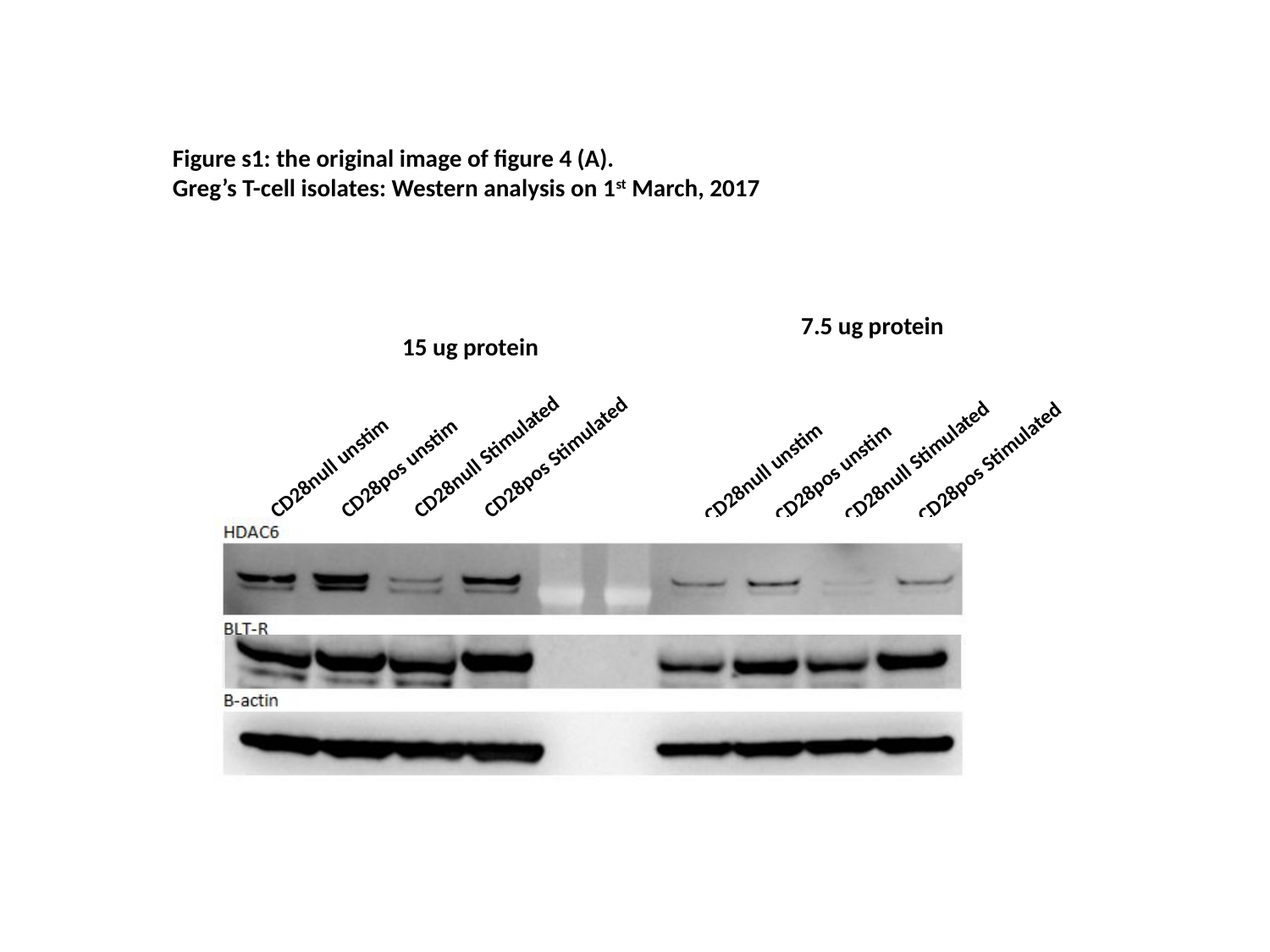

Figure s1: the original image of figure 4 (A).
Greg’s T-cell isolates: Western analysis on 1st March, 2017
7.5 ug protein
15 ug protein
CD28null unstim
CD28pos unstim
CD28null Stimulated
CD28pos Stimulated
CD28null unstim
CD28pos unstim
CD28null Stimulated
CD28pos Stimulated
